# STR profiling and Copy Number Variation analysis on single, preserved cells using current Whole Genome Amplification methods

**DOI:** 10.1038/s41598-017-17525-5

**Published:** 2017-12-07

**Authors:** Ann-Sophie Vander Plaetsen, Lieselot Deleye, Senne Cornelis, Laurentijn Tilleman, Filip Van Nieuwerburgh, Dieter Deforce

**Affiliations:** 1 Laboratory of Pharmaceutical Biotechnology, Ghent University, Ottergemsesteenweg 460, 9000 Ghent, Belgium; 2 Department of Life Science Technologies, imec, 3001 Leuven, Belgium

## Abstract

The growing interest in liquid biopsies for cancer research and cell-based non-invasive prenatal testing (NIPT) invigorates the need for improved single cell analysis. In these applications, target cells are extremely rare and fragile in peripheral circulation, which makes the genetic analysis very challenging. To overcome these challenges, cell stabilization and unbiased whole genome amplification are required. This study investigates the performance of four WGA methods on single or a limited number of cells after 24 hour of Streck Cell-Free DNA BCT preservation. The suitability of the DNA, amplified with Ampli1, DOPlify, PicoPLEX and REPLI-g, was assessed for both short tandem repeat (STR) profiling and copy number variant (CNV) analysis after shallow whole genome massively parallel sequencing (MPS). Results demonstrate that Ampli1, DOPlify and PicoPLEX perform well for both applications, with some differences between the methods. Samples amplified with REPLI-g did not result in suitable STR or CNV profiles, indicating that this WGA method is not able to generate high quality DNA after Streck Cell-Free DNA BCT stabilization of the cells.

## Introduction

In multiple fields of life sciences, single cell analysis is crucial to study the heterogeneity of cell populations or when only a limited number of cells is available^1^. This is applicable for liquid biopsies in cancer research, cell-based non-invasive prenatal testing (NIPT) and preimplantation genetic diagnosis (PGD). In the context of liquid cancer biopsies and cell-based NIPT, intact cells are extremely rare and fragile in the peripheral circulation^2,3^.

Considering a single human diploid cell contains only 6–7 pg of genomic DNA, whole genome amplification (WGA) is required to increase the amount of DNA up to ng- or µg-level when multiple genetic analyses or MPS are to be performed on a single cell. According to the manufacturer’s instructions, current MPS library preparation kits require a DNA input as low as 1 ng for Nextera XT library preparation and 500 ng for standard Illumina PCR-free library preparation. Recently, several research groups have been studying the performance of different WGA methods for specific applications. As some WGA methods only perform well in certain applications, an overall best performing WGA method does not exist^4–11^. The suitability of a WGA method depends on the intended downstream application. Studies demonstrate that PCR-based methods result in a more balanced genome coverage, which makes them more suitable for CNV analysis, while multiple displacement amplification (MDA) based methods are preferred for single nucleotide polymorphism (SNP) detection^4^.

This study wants to investigate the performance of several state-of-the-art WGA methods in a setting mimicking cell-based NIPT and liquid cancer biopsy, in which single cells are isolated from a patient blood sample. Cell isolation and downstream application for genetic analysis cannot always be performed on-site and/or immediately upon blood retrieval. Therefore, the potential of transportation and longer storage without the loss of cell quality would be opportune in a clinical setting. The widely-used K_2_- or K_3_-EDTA blood tubes demand storage at 4 °C and blood processing within 6 hours after venipuncture, before nucleated blood cell lysis occurs and cell-free DNA levels increase^12^. Several alternative collection devices with cell- and/or DNA-stabilizing properties have emerged such as CellSave tubes, PAXgene tubes and Streck Cell-Free DNA BCT tubes (cfDNA BCTs). Comparative studies of their performance in the context of liquid cancer biopsy testing or cell-free NIPT, agreed that different stabilizing collection devices should be chosen according to the desired application^13–15^. The cfDNA BCTs (Streck, Nebraska, USA) appeared best for this study, since the tubes are designed to prevent lysis of nucleated blood cells and circulating epithelial cells by stabilizing these cells and thereby preventing DNA release into the plasma for up to 7 days at temperatures between 15 °C to 30 °C^13,15^. In contrast to most fixatives, cfDNA BCT reagent claims to be a formalin-free preservative^16,17^. Formalin is known for its damage to DNA through the introduction of chemical modifications such as DNA protein denaturation, protein-DNA cross-linking and methylation of nucleic acids, which influences downstream genetic analyses^18^. This formalin-free fixative might stabilize and thereby preserve desired cells without decreasing the DNA quality. As WGA performance depends on the input DNA quality, fixation might also influence the WGA. However, the exact effect on the amplification may possibly differ between the WGA methods.

In this study, the influence of 24 hour Streck Cell-free DNA BCT® preservation on four different WGA methods was determined. Samples consisting of 1 or 3 cells, in triplicate, were collected from a lymphoblastoid Loucy cell line after 24 hour preservation. This lymphoblastoid cell line is representative for most human cells and is valuable for CNV detection performance studies, since it shows some known aneuploidies and CNVs (≥3 Mb), as studied and documented in the COSMIC project^19^. Four WGA kits, each representing a different WGA method, were selected from earlier performance studies for CNV analysis on single, live cells^5,11^. The MDA-based WGA method, REPLI-g single cell kit (Qiagen, Hilden, Germany), uses the high-fidelity enzyme, Phi29 polymerase, which holds a proofreading activity and thereby reduces the introduction of nucleotide errors during WGA. The strand displacement activity of the polymerase creates a hyper-branched DNA structure and consequently exponential amplification occurs^20^. The PicoPLEX® WGA Kit (Rubicon Genomics Inc., MI 48108, USA) combines MDA with standard PCR amplification, utilizing self-inert degenerate primers, which causes semi-linear amplification. PicoPLEX® WGA Kit has already proven its utility in clinical settings such as PGD for the detection of CNVs^10,21^. Other PCR-based WGA methods include Ampli1™ WGA Kit (Silicon Biosystems, Castel Maggiore, Italy) and DOPlify™ WGA (Reproductive Health Science, Thebarton, Australia). Ampli1 is a ligation-adapter PCR-based WGA method, which is characterized by an initial restriction enzyme-based fragmentation of the DNA. Therefore, Ampli1 should be more robust for fragmented or degraded DNA templates. Latest developed DOPlify WGA is based on the straightforward and widely used degenerate oligonucleotide primed PCR (DOP-PCR). DOPlify is an advanced DOP-PCR with possibly new primers and new generation polymerases with high fidelity and proofreading activity^22^.

As fetal cell isolation from the maternal blood stream is still prone to maternal cell contamination, short tandem repeat (STR) profiling is often performed on each individual cell to exclude maternal contamination and confirm fetal identity^23^. CNV analysis is currently the most common downstream genetic analysis in cell-based NIPT and liquid cancer biopsy^24–27^. So, after the amplification with REPLI-g single cell kit, Ampli1™ WGA Kit, DOPlify™ WGA and PicoPLEX® WGA, both the suitability of the amplified DNA for STR genotyping and CNV detection was assessed.

## Materials and Methods

### Experimental design

**Figure 1:**
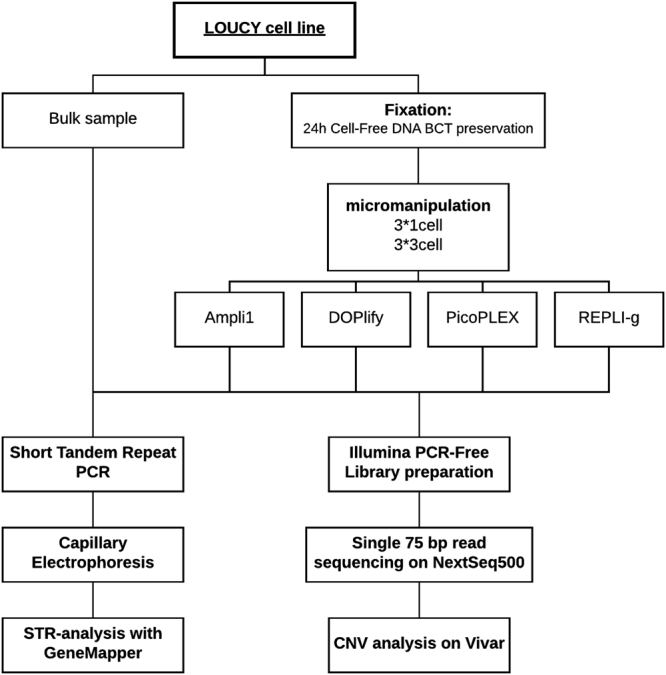
Experimental design. Cells from the Loucy cell line were preserved for 24 hours in Cell-Free DNA BCT reagent. Samples consisting of 1- or 3-cells were isolated from this fixed cell suspension for each WGA method. Ampli1, DOPlify, PicoPLEX and REPLI-g were used for amplification, followed by Illumina PCR-Free library preparation and next generation sequencing. In parallel, STR-PCR and capillary electrophoresis was performed on all samples, including a bulk sample from the cell line.

In this study, the performance of four WGA methods on a limited number of cells was examined after 24 hour cell-free DNA BCT® preservation (Fig. 1). The suitability of the DNA for STR typing and CNV analysis was studied. A cell suspension from a female lymphoblastoid Loucy cell line (DSMZ, ACC394) was transferred to a cfDNA BCT tube and stored at room temperature for 24 hours. For each WGA method, samples consisting of 1 or 3 cells were collected, in triplicate, from this fixed cell suspension, using micromanipulation^28^. In parallel, a bulk DNA sample from the cell line was obtained. This bulk DNA serves as a reference/golden standard to allow impartial conclusions when comparing the WGA methods. The 1- and 3-cell samples were amplified using four different WGA methods. Subsequently, human identification STR analysis, PCR-free Illumina library preparation and massively parallel sequencing (MPS) were performed on all WGA products. STR typing was also performed on the bulk DNA, in parallel, which was used as a reference profile. A reference 180 K arrayCGH profile (Agilent Technologies) from unamplified DNA of a bulk sample of the cell line was available from a previous study (Supplementary Figure S1)^5^. The following reference aneuploidies and CNVs (≥3 Mb) were present: a deletion of an entire X-chromosome, two deletions of ±45 Mb (consists of 6 Mb and a 36.5 Mb deletion interspersed by a 2.5 Mb normal ploidy region) and 30 Mb on respectively 5q14.3-q31.1 and 5q33.1-q35.3, a deletion of ±60 Mb on 6q21-q27, a deletion of 3 Mb on 12p13.31-p13.2, a ±26 Mb duplication of 13q31.3-q34, and two deletions of 16 Mb and 3 Mb on respectively 16p13.3-p13.11 and 16q24.2q24.3. CNV calling accuracy after WGA was evaluated as described by Deleye *et al*.^11^ by comparing the CNV calls in all WGA samples with those called in the earlier sequenced bulk DNA sample using 1 Mb windows (Supplementary Figure S2).

A positive control containing 1 µL of high quality male control DNA (Human Genomic DNA, Roche, 100 µg (500 µl)) at a concentration of 30 pg/µL was included during each WGA method. This control sample was used to evaluate the performance of the WGA kit, without the influences of cell isolation, cell lysis and DNA extraction. A negative control, consisting of 1 µL H_2_O, was added to detect contamination introduced during the WGA.

A positive control containing saliva was included during multiplex STR-PCR to verify the success of the PCR reaction. A no template control (NTC) sample, consisting of H_2_O, was included to rule out any non-specific amplification during STR-PCR.

### Cell culture and isolation

The cells from the lymphoblastoid Loucy cell line (DSMZ, ACC394) were grown in Roswell Park Memorial Insititute (RPMI-1640) medium (Life technologies™, Carlsbad, USA). This cell medium was supplemented with 10% fetal bovine serum (Life Technologies, Carlsbad, USA) and a mix of penicillin at 100 units/mL and streptomycin at 100 µg/mL (Life technologies™, Carlsbad, USA). Cells were cultured at a temperature of 37 °C and a 5% CO_2_ level. A Phosphate-buffered saline (PBS) suspension containing 1 * 10^6^ cells in 10 mL was transferred to a cfDNA BCT tube and stored at room temperature for 24 hours. A known number of cells was isolated, using micromanipulation, in the same manner as previously described by Deleye *et al*.^11^. A bulk DNA sample from the Loucy cell line was prepared using the DNeasy Blood & Tissue kit (Qiagen, Hilden, Germany) on ±5 * 10^6^ cells.

### Whole genome amplification

Cell lysis and amplification with Ampli1™ WGA Kit (Silicon Biosystems, Castel Maggiore, Italy), DOPlify™ WGA (Reproductive Health Science, Thebarton, Australia), REPLI-g Single Cell Kit (Qiagen, Hilden, Germany), and PicoPLEX® WGA Kit (Rubicon Genomics Inc., MI 48108, USA) were performed according to manufacturer’s recommendations. The amplified DNA was purified using the Genomic DNA Clean & Concentrator kit (version 1.0.0, Zymo Research, Irvine, USA) using 5x binding buffer following manufacturer’s instructions. DNA concentration was determined using the Qubit dsDNA High Sensitivity Assay kit (Life technologies™, Carlsbad, USA).

### STR genotyping

The purified DNA samples served as template for the in-house multiplex STR-PCR assay, based on the Promega Powerplex. This multiplex PCR was used to simultaneously amplify the Amelogenin locus and 14 tetrameric STR loci across the human genome: D3S1358, TH01, D21S11, D18S51, vWA, D8S1179, TPOX, FGA, D5S818, D13S317, SE33, CD4, D7S820, and D16S539. The total volume in each reaction mix was 50 µL, containing 2.5 U Hotstar Taq polymerase (Qiagen, Hilden, Germany), 0.5 mM MgCl_2_ (Qiagen, Hilden, Germany), 0.4 µg/µL albumin (Sigma-Aldrich, Saint Louis, USA), 1x PCR buffer (Qiagen, Hilden, Germany), 0.15 µM − 1 nM of each primer and 30 µL of purified DNA. Supplementary Table S1 shows the exact concentrations used for each primer. Respectively 1 ng, 1 ng, 5 ng and 4 ng purified DNA from Ampli1, DOPlify, PicoPLEX or REPLI-g amplification product was added to the reaction mixture. The multiplex PCR was performed in a SimpliAmp Thermal Cycler (Life Technologies, Carlsbad, USA) with an initial denaturation step at 95 °C for 15 min, followed by 28 amplification cycli of 94 °C for 1 min, 58 °C for 1 min and 72 °C for 1 min 20 s. A final elongation step at 72 °C for 10 min was added. As a positive control, 30 µL of a 1:10 dilution of saliva was used, whereas for the non-template control the sample was replaced by 30 µL of H_2_O.

### STR genotyping analyses

STR profiles for all samples, including the bulk DNA sample, were generated with capillary electrophoresis. Separation and analysis of the amplified PCR fragments was performed using the ABI 3500 Genetic Analyzer equipped with GeneMapper ID-x 1.2 software (Applied Biosystems™, Carlsbad, USA) following manufacturer’s recommendations. A detection threshold of 50 RFU was used to indicate allele peaks. The total dropout rate (DO%) was assessed for all samples and compared between the four WGA methods as well as between the 1- and 3-cell samples per WGA method. The dropout rate was calculated based on the following formula:

$$[1-(\frac{total\,number\,of\,observed\,alleles}{total\,number\,of\,expected\,alleles})]\times 100 \% $$

### TruSeq DNA PCR-free HT library preparation

Fragmentation of all WGA products to a mean size distribution of 350 bp was performed using the S2 Focused Ultrasonicator with Adaptive Focused Acoustics (AFA) technology (Covaris, Woburn, USA), according to manufacturer’s instructions. Resuspension buffer (provided in the TruSeq DNA PCR-free kit) was used to dilute all samples to a volume of 52.5 µL with a DNA input between 350 ng and 1 µg. Subsequently, library preparation, library quantification and single-end indexed 75 bp sequencing was performed as earlier clarified by Deleye *et al*.^11^, respectively using the TruSeq DNA PCR-free HT library preparation kit (Illumina, San Diego, USA), the Sequencing Library qPCR Quantification kit (Illumina, San Diego, USA) and a high-output NextSeq. 500 flow-cell (Illumina, San Diego, USA).

### CNV data analysis and statistical analysis of the read count variance

CNV data analysis was performed as described by Deleye *et al*.^11^ using the ViVar Software^29^. The CNV calls were detected based on the QDNAseq algorithm. The results were analyzed using 1 Mb windows. The CNV profiles are visualized as line plots in which the windows are represented as dots, ordered based on their genomic position, as indicated by the x-axis. The horizontal lines refer to the segments. The alternating white and gray background identifies the chromosomes while the y-axis shows the median normalized log_2_ transformed per-window read counts. Raw sequencing data are deposited in the NCBI Sequence Read Archive under project accession number PRJNA397729. On ViVar they are available under the project: Streck (https://holmes.ugent.be:9090/vivar/).

The average read count variance between the windows across the genome was calculated as described by Deleye *et al*.^11^. The following formula was used: $\frac{{\sum }_{i}^{N-1}{((\frac{{x}_{i}}{a})-(\frac{{x}_{i+1}}{a}))}^{2}}{N}$ in which ‘*N’* is the number of windows, ‘*x*_*i*_’ the read count in window *i*, ‘*x*_*i*+*1*_’ the read count in the next window *i* + 1 and *‘a’* the median number of reads across all windows. In this formula, the read count in each window was scaled by factor ‘a’, normalizing the result for the total number of reads that was sequenced for the sample. To test for significant differences of this metric between groups of samples, a Kruskal-Wallis H test was performed. This way, following groups of samples were compared: Ampli1 *versus* DOPlify *versus* PicoPLEX for 1 cell samples (N = 3), for 3 cells samples (N = 3), and for the 1 cell and 3 cells samples taken together (N = 6). Comparisons with p-values smaller than 0.05 were considered statistically significant.

### True and false positives

All genomic line profiles were checked for accurate variants. Deletions or duplications in sample CNV profiles that were also called in the reference CNV profile from the bulk DNA sample, were defined as true positives. CNV calls that are not present in the reference CNV profile were labelled as false positives. The results were compared using 1 Mb windows.

## Results

### Dropouts in STR profiles

**Figure 2:**
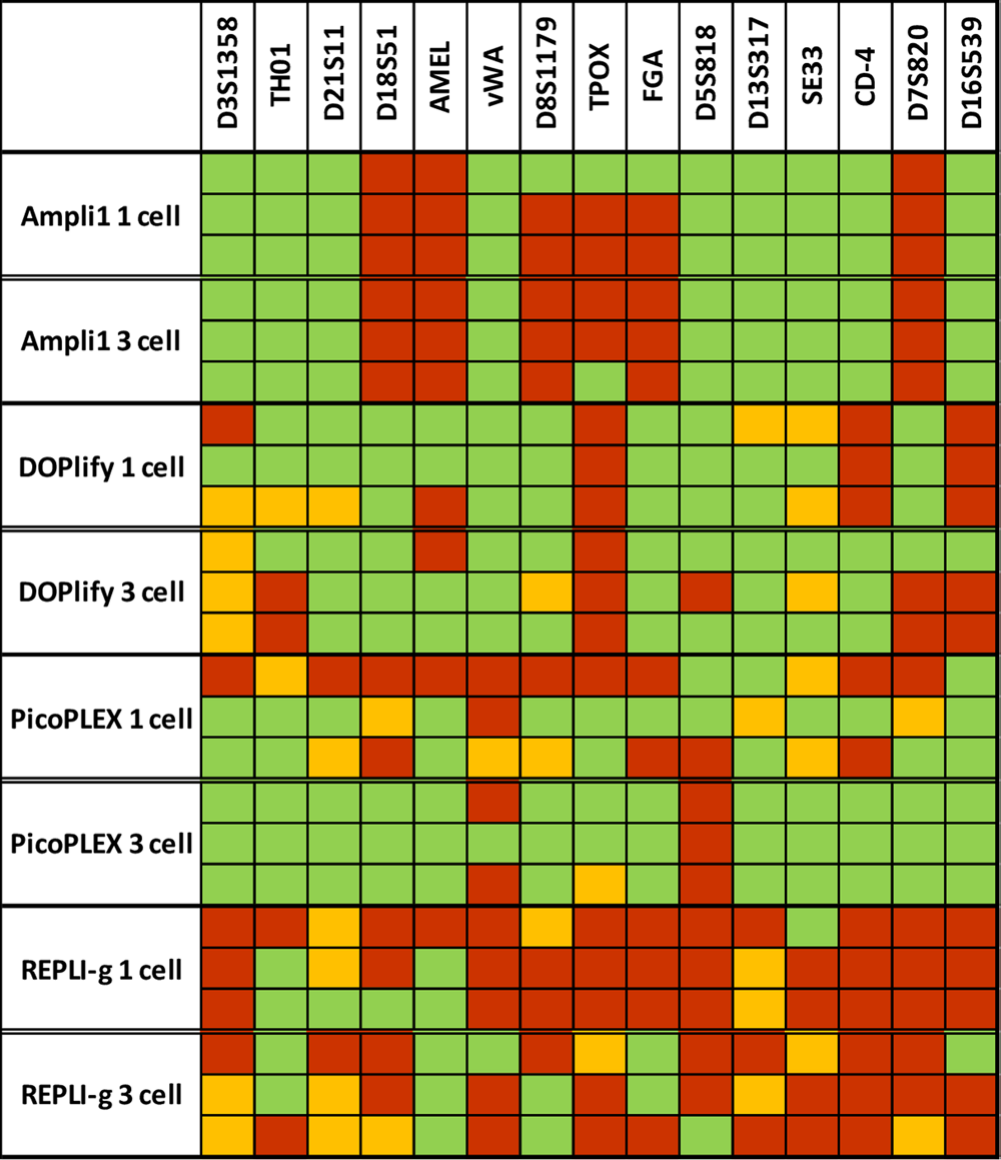
Overview of dropouts in STR profile for each WGA method. This figure illustrates which loci were called in the 1- and 3-cell samples for each WGA method. Correctly called loci are indicated in green. The orange squares represent allele dropouts, whereas a complete locus dropout is illustrated in red.

STR profiles of the amplified samples were compared with the reference STR profile from the cell line (Supplementary Figure S3). Figure 2 illustrates which loci were called in the 1- and 3-cell samples from all four WGA methods. A locus is called correctly if indicated in green. Orange squares represent allele dropouts in which only one allele of a heterozygous locus is called. A complete locus dropout is illustrated in red.

Ampli1 showed no allele dropouts, but locus dropouts of the Amelogenin locus and the STR loci D18S51 and D7S820 were present in all samples. A locus dropout of the loci D8S1179 and FGA occurred in 5 out of 6 samples, whereas a locus dropout of TPOX was present in 4 out of 6 samples. The STR profiles of the 1- and 3-cell samples were very similar. DOPlify showed 0–4 allele dropouts per sample, mainly for the loci D3S1358 and SE33. All DOPlify samples demonstrated a locus dropout of the locus TPOX, whereas the D16S539 locus dropout was present in all samples except one. The CD-4 locus dropout was only seen in the 1-cell samples. No other substantial differences were observed between the 1- and 3-cell samples. The STR profiling of the first 1-cell sample from PicoPLEX was considered as a failure because only 3 out of 15 loci were called correctly. The remaining PicoPLEX 1-cell samples showed some randomly distributed allele dropouts and a few locus dropouts. The dropout of locus D5S818 was present all but one sample. The 3-cell samples amplified with PicoPLEX resulted in remarkably more complete STR profiles with dropouts. REPLI-g resulted in unsatisfactory STR profiles with a maximum of 5 correctly called loci per sample.

Comparing the WGA methods based on the calculated dropout rate, DOPlify and PicoPLEX perform similar on 1-cell samples with a dropout rate of 28 ± 10.6% and 28 ± 11.3%. Ampli1 has a dropout rate of 33.3 ± 11.6%, whereas REPLI-g has the highest dropout rate of 77.3 ± 8.3%. For 3-cell samples, PicoPLEX excels with a dropout rate of 10.7 ± 6.1%, followed by DOPlify and Ampli1 with respectively 30.7 ± 14.1% and 37.3 ± 4.6%. Again, REPLI-g shows the highest dropout rate of 65.3 ± 2.3%. PicoPLEX and REPLI-g clearly have a lower dropout rate for 3-cell samples, whereas the number of input cells has a minor impact on Ampli1 and DOPlify, which even show a slightly higher dropout rate for 3-cell samples (Supplementary Figure S4).

### Variance in read counts per window across the genome

**Figure 3:**
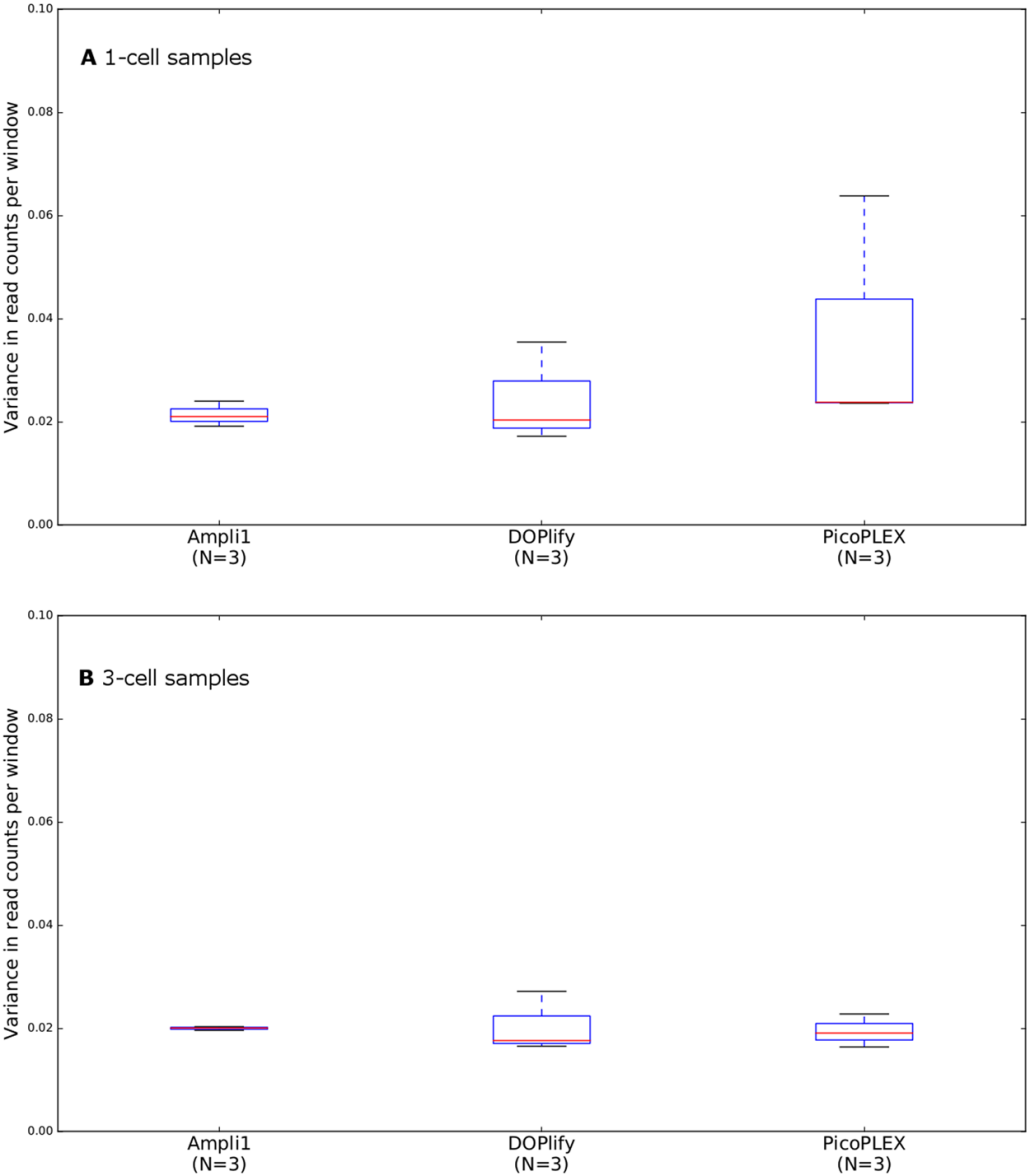
Boxplots of the average variance in read counts per 1 Mb window across the genome. (**A**) Boxplots representing all 1-cell samples for Ampli1, DOPlify and PicoPLEX (Kruskal-Wallis H test p-value = 0.511). (**B**) Boxplots representing all 3-cell samples for Ampli1, DOPlify and PicoPLEX (Kruskal-Wallis H test p-value = 0.829). REPLI-g was omitted from this figure, because it showed a very high average variance in read counts, indicating that the reads were distributed irregularly across the genome. The red line is the median variance for each WGA. The blue box contains the first and third quartile, whereas the black lines represent the minimum and maximum variance.

The variance in read counts per window across the genome gives an indication of the performance of each WGA method for CNV analysis. Only when the reads are distributed uniformly across the genome, CNVs can be called truthfully. All samples amplified with Ampli1, DOPlify and PicoPLEX showed this desired uniform distribution. Unfortunately, no conventional CNV profiles resulted from the samples amplified with REPLI-g, because the variance in read counts per window was very high, indicating the reads were distributed irregularly across the genome (Supplementary Figure S5). Therefore, no further downstream (statistical) analyses were performed for REPLI-g. Figure 3 illustrates the boxplots of the average variance in read counts per 1 Mb window across the genome for all 1-cell and 3-cell samples of Ampli1, DOPlify and PicoPLEX. Regarding the 3-cell samples, the average variance in read counts was similar within these three WGA methods. For the 1-cell samples, the average variance was also similar, but the boxplots show a bigger difference in variance between the three single cell samples, especially for PicoPLEX. One PicoPLEX single cell sample showed variance that is three times higher, compared to the other PicoPLEX samples. For the 1-cell samples (p = 0.511) and the 3-cell samples (p = 0.829), the average variance in read counts per window did not differ significantly between the different WGA methods. Moreover, including all samples per WGA method (N = 6), no significant difference was observed between the three WGA methods (P = 0.834).

### CNV analysis using a 1 Mb window

**Figure 4:**
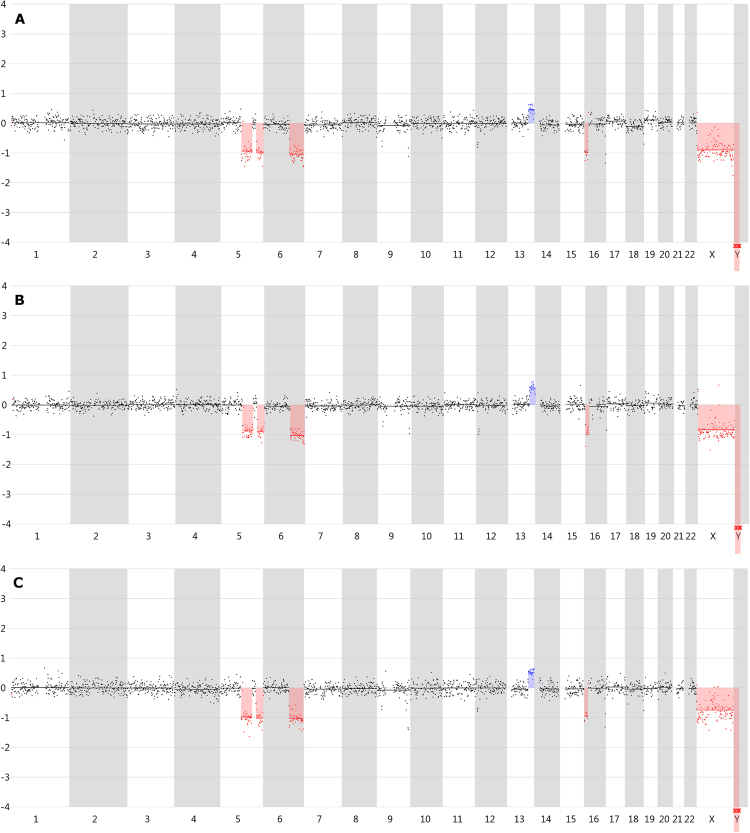
CNV line profiles generated with Vivar using 1 Mb windows. Representative CNV line profile of a (**A**) 1-cell sample amplified with Ampli1. (**B**) 1-cell sample amplified with DOPlify. (**C**) 1-cell sample amplified with PicoPLEX. Segments in red represent deletions, whereas blue segments indicate duplications. As REPLI-g did not result in reliable CNV profiles, it was omitted from this figure.

Supplementary Figure S6 demonstrates the 1 Mb window line profiles of all samples except for REPLI-g. These are also available online (https://holmes.ugent.be:9090/vivar/). As previously mentioned, REPLI-g was omitted from this CNV analysis. The CNV line profile of one representative 1-cell sample of Ampli1, DOPlify and PicoPLEX is represented in Fig. 4. The expected duplications and deletions (≥3 Mb) as present in the reference 1 Mb CNV line profile, sequenced using a bulk sample from the Loucy cell line by Deleye *et al*.^11^, were called in all WGA methods except for one 3-cell sample from PicoPLEX. This sample shows no call for the Chr13q duplication, however, higher read counts per window at this genomic position are clearly notable. In total, only extra CNVs were observed. One DOPlify 1-cell sample called an extra deletion at the end of chromosome 10, whereas one PicoPLEX 3-cell sample called an extra deletion at the beginning of chromosome 5. Three samples showed an extra deletion at the end of chromosome 16, which is also present in the reference 180 K arrayCGH profile. Supplementary Table S2 indicates the called CNVs per sample. CNV profiles from 1-cell samples showed no notable differences compared with 3-cell samples.

## Discussion

The goal of this study was to investigate the performance of four WGA methods on a limited number of cells after 24 hour of Streck cfDNA BCT preservation. Both the suitability of the amplified DNA for STR genotyping and CNV analysis was assessed. It was observed that REPLI-g is not suitable to generate high quality DNA that allows STR genotyping or CNV calling in this specific set-up. The performance of Ampli1, DOPlify and PicoPLEX, on the other hand, was considered as suitable for both applications, with some differences between the three WGA methods.

STR genotyping results in a remarkably high dropout rate for REPLI-g. Moreover, with a maximum of 5 correctly called STR loci per sample, the identity of a single cell is difficult to prove. PicoPLEX showed the lowest dropout rate and therefore performed best for STR genotyping. Still, one PicoPLEX 1-cell sample failed, and the reason for this failure was not clear. Possibly, this WGA protocol is less robust, which should be considered when choosing one over another WGA method. DOPlify and Ampli1 prove to be robust and reproducible WGA methods for STR genotyping of 1-cell and 3-cell samples but show a higher dropout rate than PicoPLEX. A consistent dropout of the same loci in each sample with Ampli1 is notable and can be related to the WGA working mechanism, as restriction enzymes are used for the initial fragmentation of the DNA. Probably, the enzymes cleave at the STR primer binding sites. This way, PCR amplification of these specific loci is inhibited and consistent locus dropout occurs. If necessary, other STR primers can be selected for the DNA typing after Ampli1 WGA.

The variance in read counts per window across the genome was similar for Ampli1, DOPlify and PicoPLEX. The uniform distribution of reads across the genome allows reliable CNV calling for these WGA methods. One single-cell PicoPLEX sample, which failed to show a descent STR profile as well, now showed a higher variance in read counts per window compared to the others. Nevertheless, the CNV profile of this sample was similar to the others, indicating that the higher variance did not impede correct CNV analysis. As already mentioned above, we attribute these negative results to a lower robustness of the PicoPLEX WGA protocol. This might sporadically lead to a biased amplification, which seems to influence STR genotyping but not CNV analysis. Samples amplified with REPLI-g did not result in CNV profiles, due to the irregular distribution of reads across the genome. The poor performance of REPLI-g might be the result of the exposure of the cells to the cfDNA BCT preservative. Possibly, this fixation reagent introduces fragmentation of the genome, which results in smaller DNA fragments. Unfortunately, it is inherent to the MDA mechanism that small input DNA fragments impede the strand displacement activity of the Phi29 polymerase, which would explain these bad results. The assumption that the bad performance of REPLI-g is caused by the cfDNA BCT reagent is supported by the study from Deleye *et al*.^11^,which demonstrates a suitable performance of REPLI-g for CNV calling on 1-, 3- and 5-cell samples. This study followed the same experimental set-up, except for the cfDNA BCT preservation step. In both studies, similar boxplots are generated for Ampli1 and DOPlify, indicating that no negative effects on the distribution of reads result from the preservation step.

Ampli1, DOPlify and PicoPLEX resulted in excellent 1 Mb window CNV line profiles with only minor differences. Ampli1 shows a 100% sensitivity and specificity, which means that all expected CNVs are called and that no false positives were present. DOPlify shows a 100% sensitivity as well, but an extra Chr10q deletion was called in one single cell sample. For this sample, a higher variance in read counts per window across the genome was noted, which might reduce the CNV calling reliability of the Vivar software and probably cause the calling of this incorrect deletion. For PicoPLEX, one sample showed a false-positive Chr5p deletion and a false-negative Chr13q duplication, which reduces the sensitivity and specificity of this method. However, the region corresponding with the Chr13q duplication clearly shows higher read counts. Compared to the other 3-cell samples from PicoPLEX with a 100% sensitivity and specificity, the average variance in read counts per window across the genome was higher, which might again reduce the CNV calling reliability of the Vivar software for this sample. Furthermore, two Ampli1 samples and one PicoPLEX sample showed an extra deletion at Chr16q, which was not considered as a false positive, since this CNV is also present in the 180 K arrayCGH reference profile from the cell line. Comparing this study with the study by Deleye *et al*.^11^, DOPlify performs very similar with or without cfDNA BCT preservation, whereas Ampli1 results in a more complete CNV profile with less incorrect calls, when 24 hour cfDNA BCT stabilization of the cells is performed.

After Streck cfDNA BCT preservation, there is no overall best performing WGA method. The most suitable method must be selected based on the intended downstream application. However, REPLI-g can be omitted from the options after cfDNA BCT fixation.

## Electronic supplementary material

Supplementary information
