# Supplementary material for: STR profiling and Copy Number Variation analysis on single, preserved cells using current Whole Genome Amplification methods
Source: Sci Rep. 2017 Dec 7;7:17189. doi: 10.1038/s41598-017-17525-5 (PMC5719346; doi:10.1038/s41598-017-17525-5)
Supplement: Supplementary file 1 — Supplementary information [file 41598_2017_17525_MOESM1_ESM.pdf]

STR profiling and Copy Number Variation analysis on single, preserved cells using current Whole Genome Amplification methods

Ann-Sophie Vander Plaetsen[1,#], Lieselot Deleye[1,#], Senne Cornelis[1,2], Laurentijn Tilleman[1], Filip Van Nieuwerburgh[1,†,*], Dieter Deforce[1,†]

[1]Laboratory of Pharmaceutical Biotechnology, Ghent University, Ottergemsesteenweg 460, 9000 Ghent, Belgium
[2]Department of Life Science Technologies, imec, 3001 Leuven, Belgium.
[#] These authors contributed equally.
[*]Corresponding author (email: Filip.VanNieuwerburgh@UGent.be).
[†]These authors jointly supervised.

Contact information:

Prof. Filip Van Nieuwerburgh
Ghent University, Laboratory of Pharmaceutical Biotechnology
Ottergemsesteenweg 460, 9000 Ghent, Belgium
email: Filip.VanNieuwerburgh@UGent.be

Supplementary Figure S1: 180 K array CGH profile from a bulk sample from the Loucy cell line. All CNVs, as detected in the female Loucy cell line with a resolution of 50 Kb, are illustrated in this profile. Deletions are indicated as red bars, whereas blue bars indicate insertions.

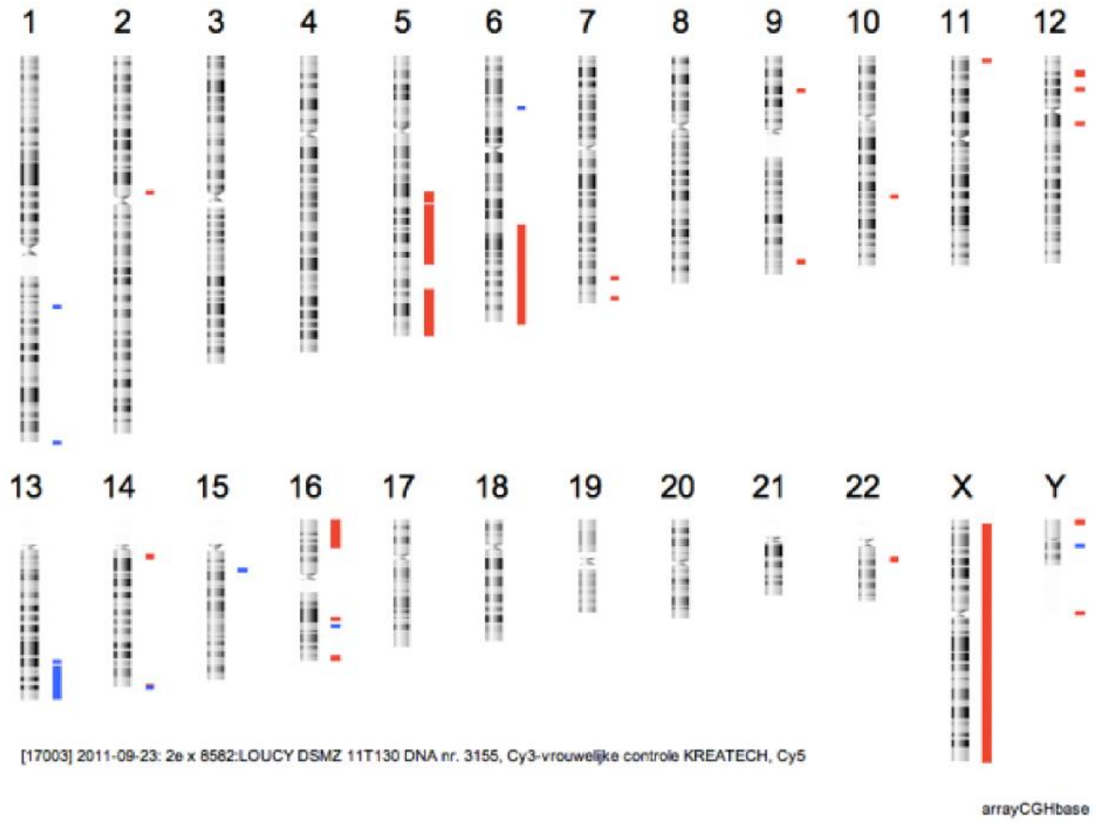

Supplementary Figure S2: 1 Mb window CNV line profile from an unamplified bulk sample from the Loucy cell line. Segments in red represent deletions, whereas blue segments indicate duplications.

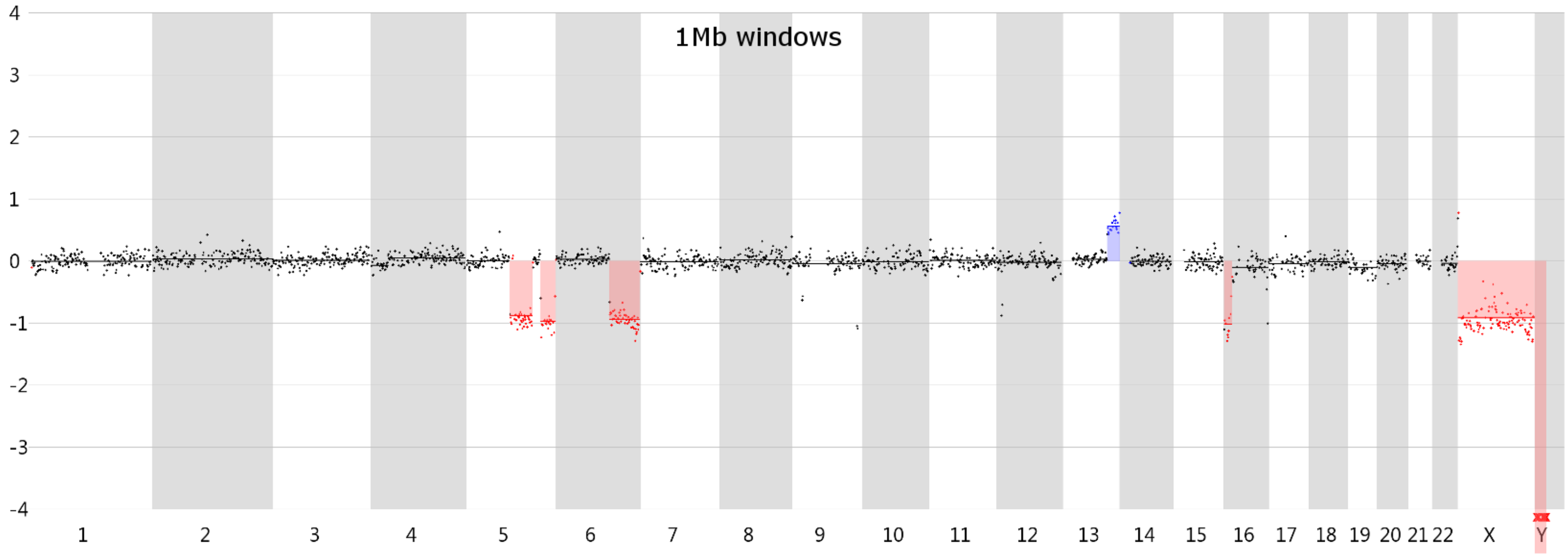

Supplementary Table S1: Concentration of the STR primers in each PCR reaction mixture.

Primer	Concentration	
SE33 F	0,5000	µM
SE33 B	1,0000	µM
D5S818 F	0,5000	µM
D5S818 B	0,5000	µM
FGA F	0,5000	µM
FGA B	0,5000	µM
D13S317 F	0,5000	µM
D13S317 B	0,5000	µM
vWA F	0,5000	µM
vWA B	0,5000	µM
D18S51F	0,5000	µM
D18S51 B	0,5000	µM
Amel F	0,5000	µM
Amel B	0,5000	µM
D21S11 F	0,5000	µM
D21S11 B	0,5000	µM
D3S1358 F	0,5000	µM
D3S1358 B	0,5000	µM
Tho1P16 F	0,5000	µM
Tho1P16 B	0,5000	µM
TPOX F	0,2500	µM
TPOX B	0,2500	µM
D7S820 F	0,6000	µM
D7S820 B	0,6000	µM
D16S539 F	0,8000	µM
D16S539 B	0,8000	µM
D8S1179 F	1,0000	µM
D8S1179 B	1,0000	µM
CD4 F	0,1500	µM
CD4 B	0,6000	µM

Supplementary Figure S3: STR profile from an unamplified bulk sample from the Loucy cell line. The Ameloginin locus and the 14 tetrameric STR loci are illustrated above the STR profile, indicating the called alleles. One peak represents a homozygous locus, whereas two peaks represent a heterozygous locus.

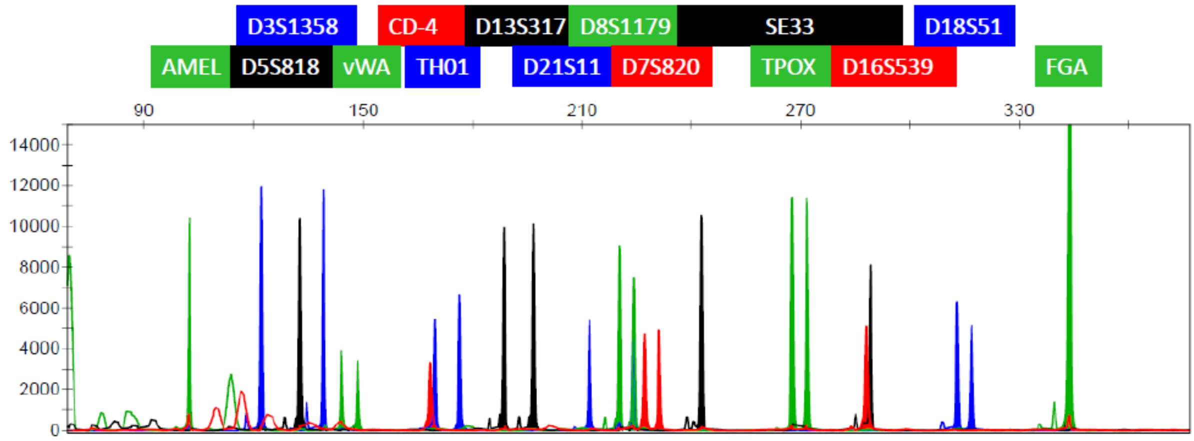

Supplementary Figure S4: Average dropout rate (%) for all 1- and 3-cell samples of the four WGA methods.

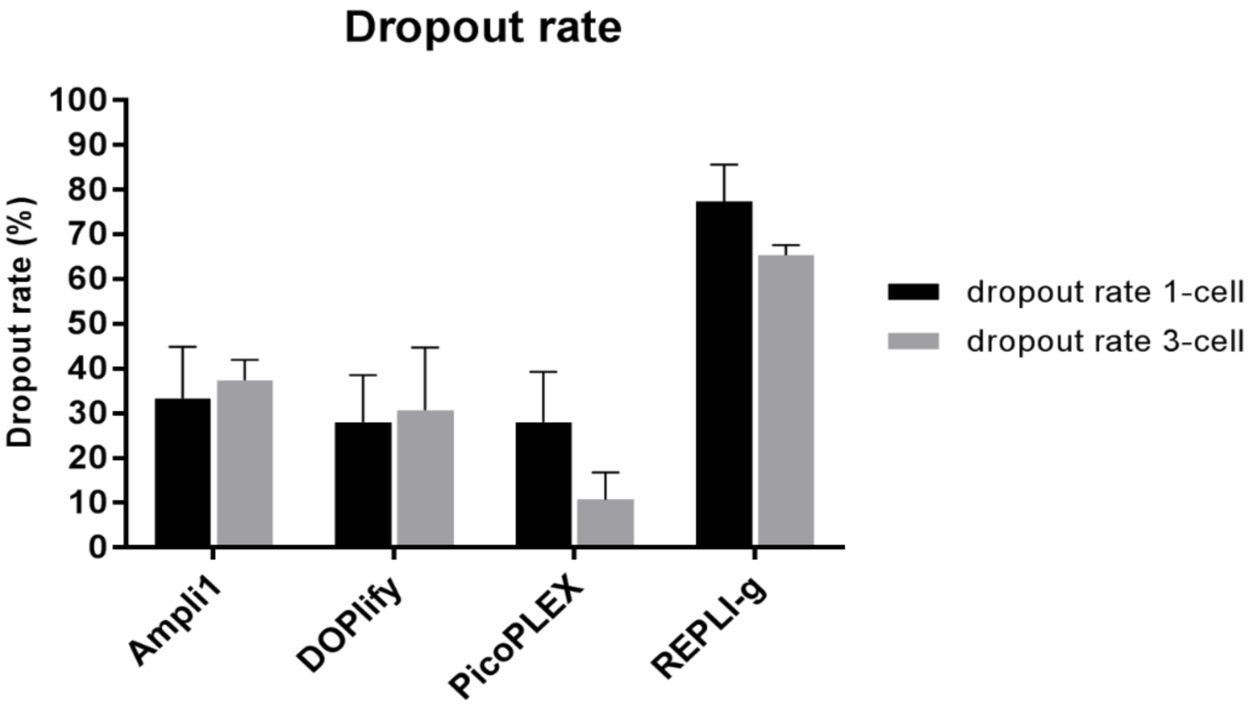

Supplementary Figure S5: 1 Mb window CNV line profiles of REPLI-g samples. Reads are distributed irregularly across the genome, leading to unusual CNV profiles.

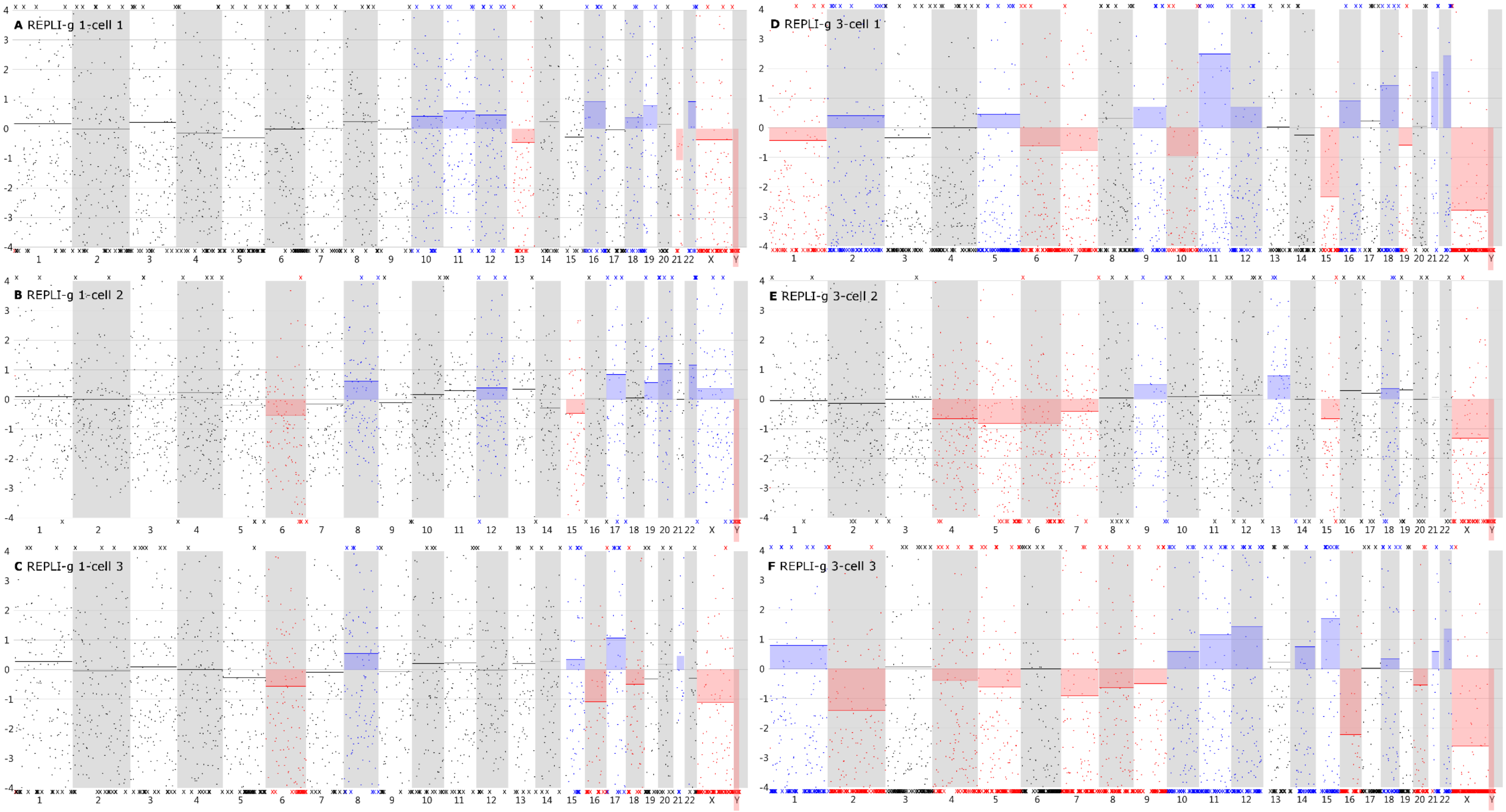

Supplementary Figure S6: 1 Mb window CNV line profiles of all samples: Ampli1 CNV profiles

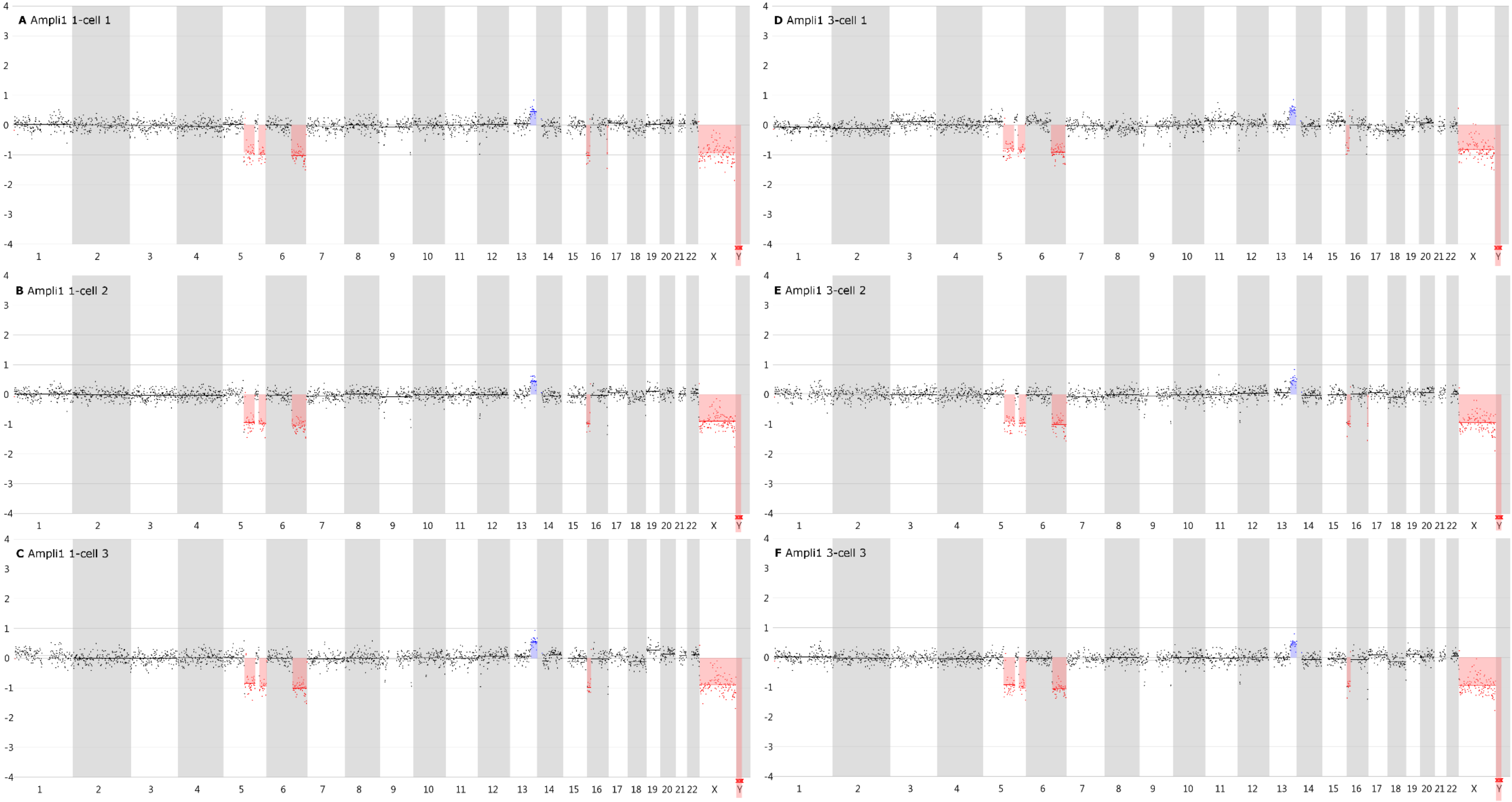

Supplementary Figure S6: 1 Mb window CNV line profiles of all samples: DOPlify CNV profiles

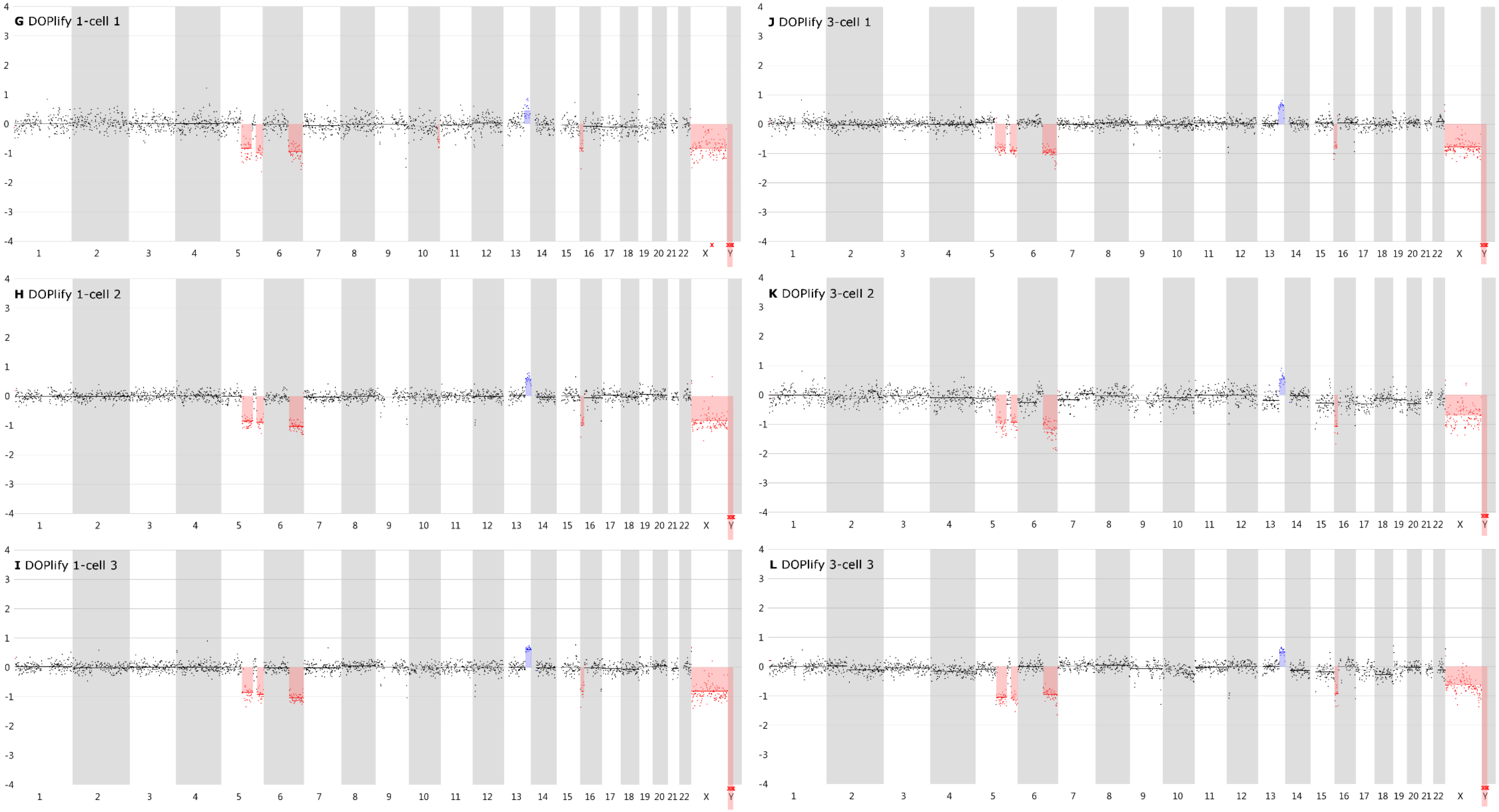

Supplementary Figure S6: 1 Mb window CNV line profiles of all samples: PicoPLEX CNV profiles

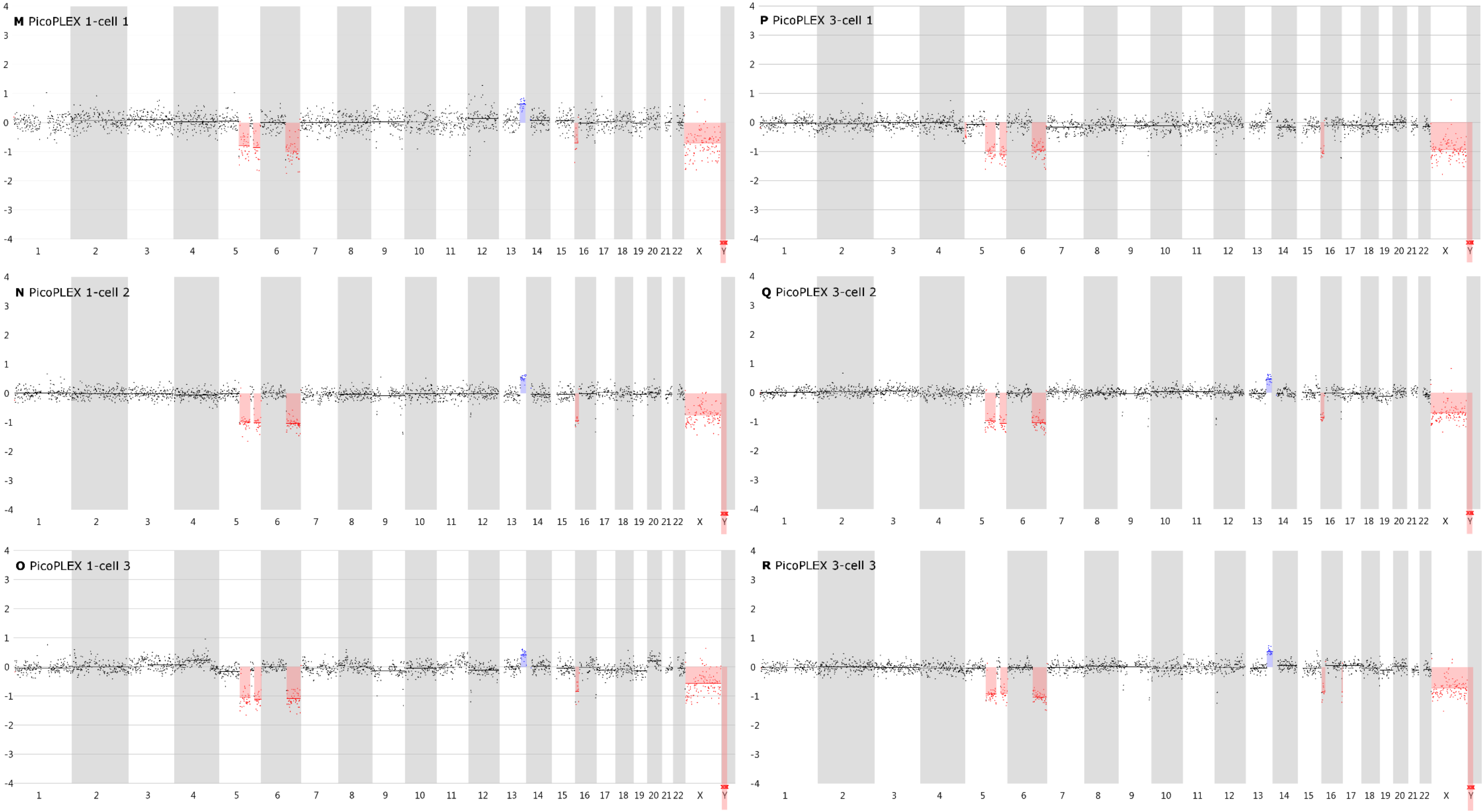

<u>Supplementary Table S2: CNVs called with Vivar using a 1 Mb window.</u> The column headers on the left side of the table show the genomic positions of all deletions or insertions (**in bold**), which are larger than 3 Mb and present in the reference 180 K arrayCGH profile. Called CNVs are indicated with a ‘v’. The column headers on the right side show the location of the incorrectly called CNVs. Subscripts (1) and (2) indicate two different CNVs that are located on the same chromosomal arm.

		Chr5q$_{(1)}$	Chr5q$_{(2)}$	Chr6q	Chr12p	**Chr13q**	Chr16p	Chr16q	X-Chr	Chr5p	Chr10q
Bulk		v	v	v		v	v		v		
Ampli-1	1c1	v	v	v		v	v	v	v		
	1c2	v	v	v		v	v		v		
	1c3	v	v	v		v	v		v		
	3c1	v	v	v		v	v		v		
	3c2	v	v	v		v	v	v	v		
	3c3	v	v	v		v	v		v		
DOPlify	1c1	v	v	v		v	v		v		v
	1c2	v	v	v		v	v		v		
	1c3	v	v	v		v	v		v		
	3c1	v	v	v		v	v		v		
	3c2	v	v	v		v	v		v		
	3c3	v	v	v		v	v		v		
PicoPLEX	1c1	v	v	v		v	v		v		
	1c2	v	v	v		v	v		v		
	1c3	v	v	v		v	v		v		
	3c1	v	v	v			v		v	v	
	3c2	v	v	v		v	v		v		
	3c3	v	v	v		v	v	v	v		
